# Silver-lining in the Time of Mayhem: The Role of Local Governments of Nepal During the COVID-19 Pandemic

**DOI:** 10.31729/jnma.5262

**Published:** 2020-11-30

**Authors:** Biplov Adhikari, Shyam Sundar Budhathoki

**Affiliations:** 1 Mulpani Primary Health Centre, Ministry of Health and Population, Kathmandu, Nepal; 2 Department of Primary Care and Public Health, School of Public Health, Imperial College London, London, United Kingdom

**Keywords:** *community responses*, *COVID-19*, *federalism*, *local governments*, *Nepal*

## Abstract

The COVID-19 pandemic has not only affected health systems but also has had deep socio-economic effects. The lockdown enforced in Nepal, had people running out of means to make ends meet, the public in fear of an unknown disease, and challenges for leaders to deliver better. Local governments of Nepal, established under the federal system, have the closest ties to the community. They have powers entrusted by the constitution to enact laws according to the needs of the community. During the 83 days of lockdown, the local governments came through for their inhabitants by managing quarantines and isolation centers, taking care of diagnostics, providing food and rations, and arranging facilities to bring back their locals stranded in other cities. The local governments improved awareness and helped maintain the lockdown. These undertakings by the local governments of Nepal highlight the importance of a community-based approach to dealing with pandemics.

## INTRODUCTION

The federal democratic republic of Nepal, a developing nation in Southeast Asia, is a country with meager resources. As of writing this article, Nepal has had 22,214 confirmed cases of COVID-19 with 70 attributed deaths.^1^

The federal structure divides the country into three tiers of governance—central, provincial, and local. Local governments form the smallest units of governance. Directly elected public representatives at the helm of the local governments have close ties to their denizens and an onus to deliver better.

In this article, we highlight the contributions of local governments in the handling of the COVID-19 pandemic in Nepal.

## STRUCTURE OF LOCAL GOVERNMENTS

Nepal adopted federalism after the promulgation of its constitution in 2015.^2^ The federal system divides the country into three tiers of governance—central, provincial, and local. Local governments form the smallest units of governance. Nepal comprises 7 provinces and 753 local governments. 6 metropolises, 11 sub-metropolises, 276 municipalities, and 460 rural municipalities represent the local levels of Nepal.^3^ The constitution has empowered each local level to function with certain autonomy in line with the central government.

Each local government has at its helm a directly elected mayor and a deputy mayor in case of municipalities or a chairperson and a vice-chairperson in case of rural municipalities. Each local level is further divided into wards, the number of wards depending upon the area and the population of individual local level. Directly elected ward chairperson and 4 ward members from the council of each ward.

All elected public representatives from within the local level form the legislative council of the local government. The constitution of Nepal 2015, grants each local level with 22 powers.^4^ These powers give the local governments the authority to enact laws in line with the laws enacted by the central government to fit the needs, situation, and resources available. The legislative council known as the 'Nagarsabha' meets twice a year to formulate and amend laws. An executive body called the 'Karyapalika' carries out the laws enacted by the legislative council.

The local levels deliver services to their inhabitants through various section offices as afforded by the constitution. A chief administrative officer leads the public servants who work in these offices to deliver the services to the inhabitants. Section offices for health and sanitation, women and child empowerment, legal matters, education, sports, planning, infrastructure development, finances, and environment deliver services in line with the laws enacted by the legislative council.

## LAWS PERTINENT TO DEALING WITH COVID-19

Among the 22 powers ensured by the constitution, the local levels can formulate their acts for basic health and sanitation and disaster management. Under the 'Basic Health and Sanitation Act', the local levels have the authority to direct resources to counteract imminent threats against the health of their inhabitants on their own accord. This made for a faster and a tailor-made approach for each local level to deal with the threat of COVID-19.

Under the act to formulate laws for disaster management, each local level has the provision to form a rapid response team. Every local level also has funds allocated for disaster management. These provisions allowed the local governments to take on the pandemic head-on, instead of having to wait for instructions and resources from the central government.

## ACTIVITIES CARRIED OUT BY LOCAL LEVELS

Nepal went into a nationwide lockdown put in place to contain the SARS-CoV-2 on the 24th March 2020.^5^ The central government enforced the lockdown in a matter of days without giving people much time to prepare. With the lockdown in place, daily wage earners found themselves without jobs, people away from home found themselves withouta way back, and local people in the angst of an unseen threat. The country had much to do in little time with scarce resources.

As the terror of the pandemic spread across the nation, the central government directed the provincial and local governments to remain vigilant of the pandemic. The central government asked local levels to prepare and allocate resources to build facilities for quarantine and isolation. Local governments responded promptly. They set up quarantine and isolation centers in a matter of days and brought the rapid response teams into action. The public representatives took it upon themselves to make their municipalities a safer place. A minor step to begin with, but something that would have taken weeks if not months for the previous centralized government to accomplish.

In the early months after the lockdown, the public fear grew disproportionately to the number of confirmed cases. Authorities could not enforce the lockdown in totality. People somehow found their way back home as the lockdown bore on without a certain end in sight. The local people, apprehensive of the returnees, turned to the public representatives. Many returnees found themselves comeback to closed gates or mobs of people labeling them as biohazards. The quarantine centers proved useful to house such returnees. Public representatives ensured that returnees completed quarantine of 14 days before integrating into the locality. Not only did that prevent the spread of the disease but also pacify the disproportionate fear stemming from the lack of knowledge and awareness.

The local governments put special emphasis on the dissemination of information regarding the disease to address the knowledge gap prevalent at the outset of the pandemic. They distributed posters and pamphlets. At the behest of local governments, the health sections paraded vehicles mounted with loud-speaks across the locale asking people to stay indoors, keep social distance, wear masks, and maintain proper hygiene etiquette including hand-washing.^6^ The health sections extensively mobilized the female community health workers to distribute posters, improve awareness, and perform active surveillance. The local governments also employed their local security forces to ensure people did not defy the lockdown.

The local governments also actively stepped up case-finding. In the beginning, only one lab under the central government performed real-time polymerase chain reaction (RT-PCR) testing. As the pandemic worsened across the world, the central government provided a rapid diagnostic test (RDT) kits to the local governments. Local health facilities tested returnees from foreign countries with RDT kits in the beginning. As more people required testing, the local governments bought the test kits out of their own pockets. When RDTs became controversial because of the high proportion of false results, local levels bought their viral transport media (VTM) for sample collection. Local levels sent collected samples to sophisticated labs under the central or provincial governments. Some municipalities even bought their PCR machines.^7^ This act of autonomy increased testing rates and active case finding across the country.

When the central government eased the lockdown and opened international borders, the repatriation of people from foreign countries began. The central government built holding centers to act as buffers to hold the repatriates before handing them over to their respective local governments. The local governments held the returnees in quarantine centers, provided them meals, performed PCR testing, and integrated them into the community after completion of their quarantine period; the local governments bore all the expenses. Some local governments found themselves overwhelmed with the management of repatriates as there were too many returnees, especially the municipalities in the Terai plains sharing the border with India.

The COVID-19 pandemic did not just strain the health system. It had pertinent social and economic ramifications. The central government began easing the lockdown from 14th June 2020.^8^ For the 83 days, during which they enforced the relatively strict lockdown, all businesses, and social ventures remained shut. Everyday wage earners and those below the poverty line were the hardest hit. Local governments took it upon themselves to provide for these people. Some local governments provided rations to the families while some opened feeding centers that provided two major meals a day.^9^ Some municipalities improvised by providing daily paying jobs for such people in projects commissioned by the municipalities themselves. That way people had food on their plates and important development projects got completed on time.

The lockdown that came into effect quickly left many who had come to urban areas for employment in a limbo. Dismissed from jobs and with no savings, people found it hard to survive away from their homes and longed to get back. But with no transportation or alternatives provided by the central government, people took radical steps. Many people walked for days on end to get back home. Rural local governments rescued many of their inhabitants by providing buses to bring them back home from the capital of Kathmandu. Some local governments helped people cross their borders by arranging vehicles for them. While civil society heavily criticized the central government for its apathy towards the plight of its citizens, the local governments earned much credit for coming to the people's aid during this act of dereliction by the central government.^10^

**Table 1:** Summary of Activities conducted by the Local governments of Nepal.

1.	Running quarantine and isolation centers.
2.	Keeping detailed records of returnees from outside the community (including international repatriates).
3.	Conducting programs to improve awareness regarding the disease.
4.	Conducting testing and sample collection out of their budget.
5.	Providing rations and running feeding centers.
6.	Creating daily paying jobs for the poor.
7.	Arranging transportation to bring back the stranded.

## LIMITATIONS FACED BY THE LOCAL GOVERNMENTS

It's not been easy for the local governments. Local governments have a limited budget earned through the collection of revenues and taxes and the toll of the pandemic has been different for each local government. The local levels in the Terai region have been the hardest hit. Returnees from India overwhelmed the local governments' efforts to manage quarantine centers. Poorly managed and overcrowded quarantine centers along with limited testing facilities compounded the problems for such municipalities and cases soared at local levels sharing their borders with India.^11^ The central and provincial governments seem to have done little to come to their aid.

There also remains the question of financial transparency. Government bodies have been infamous in the past for financial irregularities along with bribery and corruption. The local governments had to work expeditiously to buy necessary provisions for disease control. Local governments still have the obligation to show to the public that they followed due procedures. Financial irregularities regarding equipment procurement have recently defamed the central government.^12^ It will require a prompt and untampered financial and social audit to verify any wrongdoings on the local governments' part.

## IMPLICATIONS ON THE GLOBAL SCALE

Enormous distance between the government and citizens can impair service delivery, especially during times of crisis. The roles of local governments and the community in overcoming disasters of national scale are pivotal. The government of China mobilized community governance systems (CGS) to address the problems brought by the COVID-19 outbreak, including the suddenly placed lockdown and overwhelmed health systems.^13^ The Ebola and HIV outbreaks in African countries taught similar lessons to actively engage community-level systems in disease mitigation.^14^ The communities which are best represented by the local governments in the case of Nepal actively took part in disease mitigation and control. Community ownership prevented the disease from spreading across the community.

Active engagement of the community is essential to address pandemics in rural and resource-poor countries. In countries where the reach of the central government is poor, local governments can play a crucial role in creating awareness and mobilizing available resources. There is a need to arm communities and local governments across the globe with adequate resources to fight pandemics at the ground level. Local governments and communities across the world require facilitation, not instructions to come up with their customized plans to address the threat of the COVID-19 pandemic. Federal structure with a more decentralized approach as in the case of local governments of Nepal helps manage pandemic diseases.

## WAYS FORWARD

Local governments of Nepal have played an important role in the management of COVID-19 at the community level. They made significant contributions in addressing gaps not only in the health system but also the economic and social impacts caused by the pandemic. Local governments should be further strengthened with necessary resources during such times of crisis so that a prompt and robust response can begin at the community level that can be availed by everyone.

## Conflict of Interest

**None.**
